# Advancing cancer research through organoid technology

**DOI:** 10.1186/s12967-024-05824-1

**Published:** 2024-11-08

**Authors:** Guolong Zeng, Yifan Yu, Meiting Wang, Jiaxing Liu, Guangpeng He, Sixuan Yu, Huining Yan, Liang Yang, Hangyu Li, Xueqiang Peng

**Affiliations:** 1 Department of General Surgery, The Fourth Affiliated Hospital, China Medical University, Shenyang, 110032 China; 2 Shenyang Clinical Medical Research Center for Diagnosis, Treatment and Health Management of Early Digestive Cancer, Shenyang, China

**Keywords:** Tumor organoids, In vitro modeling, Tumor microenvironment, Multi-omics analysis, Gene editing, Precision medicine

## Abstract

The complexity of tumors and the challenges associated with treatment often stem from the limitations of existing models in accurately replicating authentic tumors. Recently, organoid technology has emerged as an innovative platform for tumor research. This bioengineering approach enables researchers to simulate, in vitro, the interactions between tumors and their microenvironment, thereby enhancing the intricate interplay between tumor cells and their surroundings. Organoids also integrate multidimensional data, providing a novel paradigm for understanding tumor development and progression while facilitating precision therapy. Furthermore, advancements in imaging and genetic editing techniques have significantly augmented the potential of organoids in tumor research. This review explores the application of organoid technology for more precise tumor simulations and its specific contributions to cancer research advancements. Additionally, we discuss the challenges and evolving trends in developing comprehensive tumor models utilizing organoid technology.

## Introduction

**Fig. 1:**
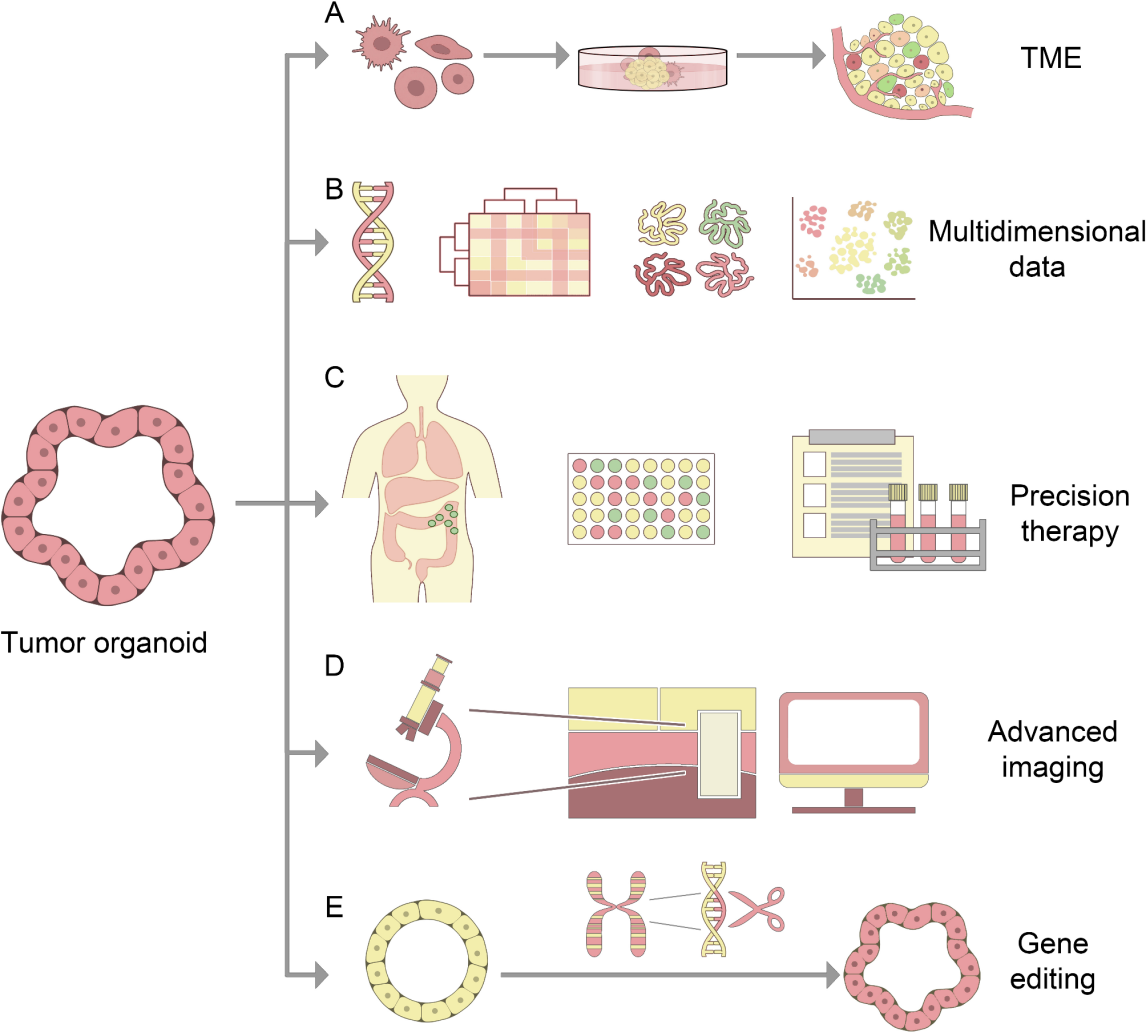
Potential applications of tumor organoids in cancer research: (**A**) To simulate the tumor microenvironment in vitro, the microenvironment conditions are replicated, and the interaction between tumor cells and various cell types in the surrounding microenvironment is investigated. (**B**) Relevant data from multidimensional sources, including the analysis and extraction of information from multi-omics data such as genomics, transcriptomics, proteomics, and monocytosis is integrated into organoids. (**C**) Tumor organoids can be utilized for tumor modeling, enabling prospective drug sensitivity testing and prediction of drug response to achieve precision treatment through the establishment of a biobank. (**D**) Tumor organoids, when combined with advanced imaging, enhance the precision and efficiency of cancer research. (**E**) The application of gene editing tools involves targeted modification of genes in organoids and the introduction of specific gene or pathway changes to study the occurrence and development of tumors and tumor modeling

As one of the major health threats, cancer continue to have high morbidity and mortality rates. Despite significant advances in the understanding of tumor mechanisms, standard cancer treatments have made only limited progress [1]. In this context, in vitro and in vivo experimental models, such as cancer cell lines, animal models, and patient-derived tumor xenografts (PDTX), have significantly advanced tumor research [2, 3]. However, there are many limitations in these models. Traditional cancer cell lines are cultured in a two-dimensional environment, which limits their ability to mimic the natural growth pattern and behavior of tumor cells in 3D space [4]. Similarly, long-term culture can lead to genetic drift, resulting in a loss of heterogeneity and failing to fully represent the complex biology of human tumors [5]. Animal models and PDTX models are significantly different from humans in genetic, physiological, and metabolic aspects, which are easily restricted by species differences and ethical issues [6, 7]. Moreover, cancer cell lines have several disadvantages, such as the absence of the tumor’s complex surrounding environment, challenges in establishing repositories, and the significant economic and time costs associated with experimental animal studies [8, 9]. Given these challenges, developing in vitro models that can more accurately simulate the characteristics of human tumors in vivo has become a crucial need in cancer research [10]. In this context, the development of in vitro models that can more precisely replicate the characteristics of human tumors in vivo has become a crucial focus in cancer research. Tumor organoids, emerging as a pivotal research area, are gaining widespread attention and are being increasingly utilized in various aspects of tumor research (Fig. 1).

Organoids, cultured in vitro, are 3D microstructures capable of self-organization and renewal, enabling them to replicate the essential functions, structure, and biological intricacies of organs. Derived primarily from induced pluripotent stem cells (iPSCs) or tissue-derived cells (TDCs), these organoids encompass various cell types, such as normal stem/progenitor cells, differentiated cells, and cancer cells [11, 12]. By utilizing patient-derived primary human cancer tissue to develop patient-derived tumor organoid (PDTO) models in vitro under 3D cultivation conditions, these organoids effectively simulate the growth of the primary tumor microenvironment in patients. This model can simulate tumor samples with precise spatial structure and histomorphology, while also preserving the heterogeneity of the primary tumor to some degree [13]. These “microtumors” offer several advantages in tumor research: [1] They replicate the development and progression of cancer in vitro; [2] They retain the phenotypic traits of the original tumor; [3] They offer matched normal tissue controls for comparison; [4] They can reconstruct the tumor microenvironment; [5] They facilitate the establishment of a biological sample repository [5]. These advantages render such models highly effective as a tool to link in vitro experiments with in vivo tumor characteristics, showing great potential for research and application. At present, tumor organoids have been successfully established for various cancer types, including gastric cancer [14], colorectal cancer [15], liver cancer [16], breast cancer [17], lung cancer [18], etc. These patient-derived tumor organoids (PDTOs) have been utilized to establish biobanks and to explore tumor onset and progression, simulate the tumor microenvironment (TME), personalize treatment approaches, and integrate multidimensional data (Table 1). The significance of organoids in biomedicine is reflected in numerous aspects. Firstly, organoids can accurately replicate the physiological state of human tissues, serving as an ideal model for drug screening and toxicity testing, thereby enhancing the efficiency and precision of drug development [19]. Patient-derived organoids can also predict individual responses to drugs and personalized treatments on a large scale. Particularly, the synergy between organoid technology and microfluidic organ-on-a-chip systems has further accelerated the translational application of organoids [20, 21].Moreover, organoids play a crucial role in disease modeling, especially for genetic disorders that are challenging to model in vitro. By incorporating genome editing technologies, organoids can more authentically simulate the pathophysiological characteristics of such diseases [22, 23].

**Table 1:** Organoids can be cryopreserved and biobanked for future research

Cancer types	Success rate	Source	Content	Reference
Gastric cancer	50%	Surgey	Whole exome and transcriptome sequencing, drug screening, personalized medicine	[14]
Colorectal carcinoma	90%	Surgey	Whole exome sequencing, drug screening, personalized medicine	[15]
Hepatocellular carcinoma	-	Surgey	Histological analysis, exploration of drug resistance mechanisms, personalized medicine	[16]
Breast cancer	80%	Surgey	Genome sequencing, CRISPR/ Cas9-mediated gene editing, high-throughput drug screening, personalized medicine	[17]
Lung cancer	13% ~ 78%	Surgey and bronchoscopic biopsy	Multi-omics analysis, niche dependent analysis and CRISPR-Cas9-based genetic engineering, molecular and phenotypic analysis	[18]
Glioblastomas	91.4%	Surgey	Personalized medicine	[146]
Bladder cancer	70%	Surgey	Deep targeted sequencing to explore tumor evolution and predicting drug response	[147]
Pancreatic cancer	75% and 83%	Fine needle aspiration (FNA) endoscopic biopsy	Simulating tumor progression	[91]

The formation of organoids can be divided into several key stages. The initial phase involves the creation of simple cell aggregates, followed by the regulation of cell differentiation and self-assembly to develop more complex three-dimensional structures. With the introduction of biomaterials and bioengineering techniques, organoids can more precisely replicate the in vivo environment [24, 25].In recent years, the integration of organoid technology with chip technology has created high-fidelity model systems. Through continuous technological innovation and advancements in analytical capabilities, organoids have evolved from initial laboratory research tools into biomedical platforms with tangible practical applications [20, 26, 27]. In this review, the latest research advancements in the use of tumor organoid models are summarized, including TME simulation, modeling of special tumor organoids, tumor mutation and tumorigenesis based on gene editing, and precision medicine. The efforts involving tumor organoids in imaging and analysis technology have also been discussed, along with the ways multi-dimensional data integration can deepen the understanding of tumor complexity. These developments not only enhance our comprehension of tumor biology but also offer a powerful research platform for the development of novel anti-tumor therapies.

## Tumor modeling: organoids assist in the simulation of tumor microenvironment

**Fig. 2:**
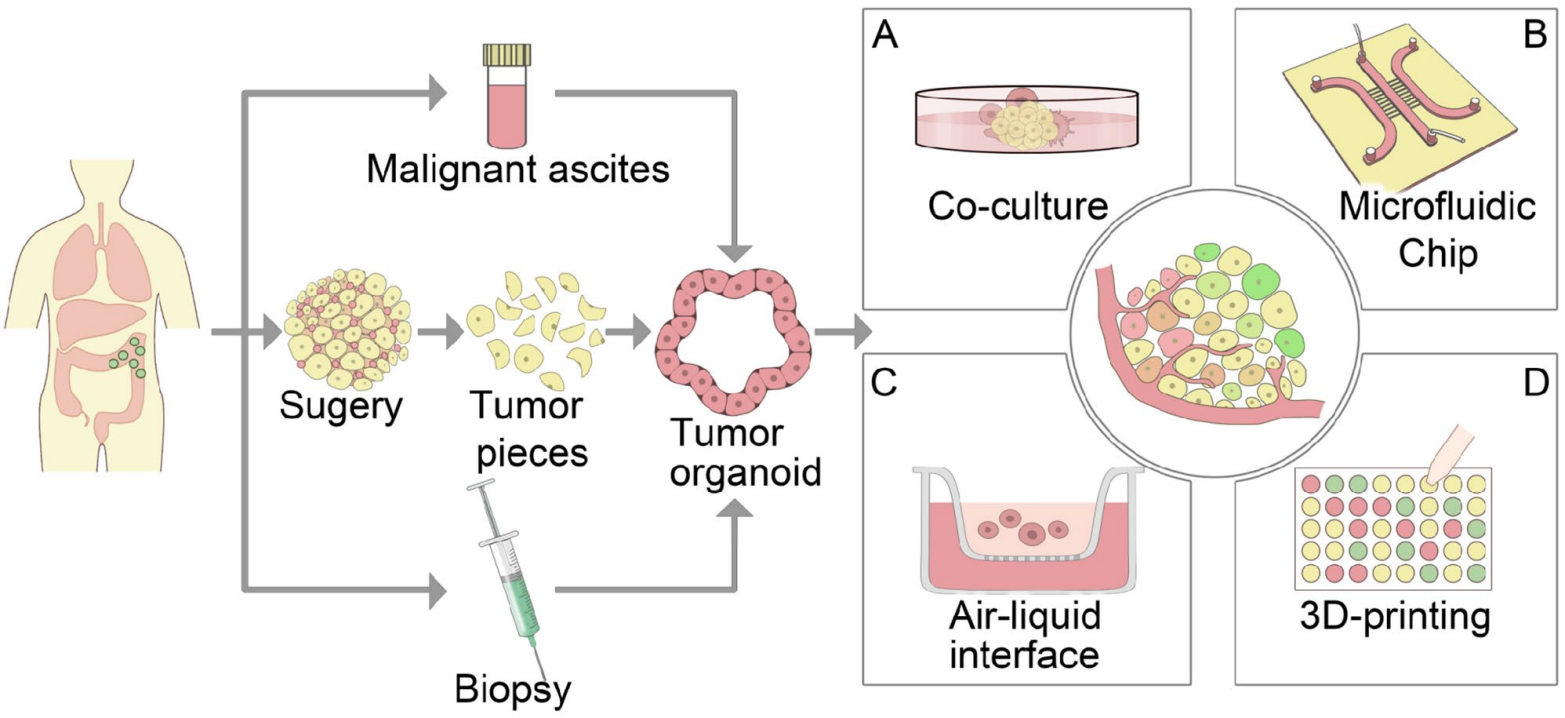
Cell sources of tumor organoids include surgical samples, needle biopsy tissues, and malignant ascites. The constructed tumor organoids can be introduced into different platforms to mimic TME: (**A**) Co-culture model: exogenous stromal cells from the tumor micro-environment; (**B**) The air-liquid interface: when cultured in the Transwell chamber, an air-liquid interface can be formed, which can reproduce the tumor immune microenvironment. (**C**) Microfluidicchip: controls the changes of environmental conditions and nutrient delivery in the TME, and reconstructs the fluid characteristics, mechanical tensile force, immune infiltration, and other complex elements in the TME; (**D**) Bioprinting: involves the use of bioactive materials to form bionic and composite TME in vitro

The TME is a complex ecosystem that facilitates tumor growth and metastasis. It comprises various components, including tumor cells, immune cells, endothelial cells, fibroblasts, blood vessels, extracellular matrix (ECM), and more. The interactions among these diverse components within the TME play a crucial role in promoting the growth, invasion, and metastasis of tumor cells [28, 29]. In addition, TME can affect the effectiveness of cancer treatment, the sensitivity of tumor cells to chemotherapy drugs, and the efficacy of immunotherapy [30]. One of the primary challenges in cancer research is accurately simulating and reproducing the complex TME. While PDTOs preserve the cellular and molecular characteristics of in vivo tumors and effectively simulate tumor heterogeneity, they typically consist solely of epithelial cell components [31]. Even though organoids exhibit a high degree of similarity to the tumor cells of the patient from whom they were derived, their application is limited by the absence of dynamic interactions with other TME components. Consequently, reconstructing tumor organoid models that incorporate TME elements in vitro is crucial for gaining a more comprehensive understanding of the original tumor characteristics [32]. In recent years, various enhancements have been made to traditional tumor organoids to more accurately replicate the characteristics of the tumor tissue microenvironment found in vivo (Fig. 2) [31, 33].

### Co-culture strategy

Incorporating additional stromal cell components into the tumor organoid model is an effective strategy to enhance its complexity and relevance, allowing for a more realistic representation of the interactions between tumor cells and their microenvironment [34]. For instance, incorporating stromal cell components into in vitro tumor organoids can enhance the accuracy of these models in simulating tumor development. In this context, when exogenous human monocyte-derived macrophages were introduced into colorectal cancer organoid culture systems, the macrophages tended to polarize into a tumor-promoting SPP1-positive state. This finding suggests that tumor cells may play a crucial role in regulating macrophage polarization [35]. Moreover, the co-culture of pancreatic cancer cells with human umbilical vein endothelial cells not only helps sustain cancer-initiating cells but also triggers the activation of the Wnt/Notch signaling pathway through the paracrine influence of endothelial cells, rather than solely depending on exogenous stem cell growth factors [36]. These findings further confirmed the importance of the interaction between different cellular components in the TME.

Numerous cancer organoid models, incorporating various types of cancer associated fibroblasts (CAFs), have been developed to investigate the intricate interplay between cancer cells and CAFs. Employing this co-culture approach with CAFs has resulted in discernible spatial variations within the TME in these models [37]. Among these are the induction of matrix formation by CAFs and the initiation of epithelial-mesenchymal transition processes. CAFs facilitate the remodeling of the ECM within tumor organoids through cytokine secretion, matrix alteration, and the modulation of signaling pathways [38, 39]. ECM remodeling frequently manifests during the initial phases of tissue morphological changes, empowering tumor cells with enhanced migration and invasion capabilities [40–42]. Remarkably, co-culturing tumor organoids with CAFs allows for the observation of induced changes in various subtypes of CAFs within tumors, thereby resulting in the functional heterogeneity of CAFs.

The co-culture system involving immune cells and tumor organoids can serve as a testing platform, facilitating the study of immune components within the TME and guiding precision cancer immunotherapy [43, 44]. Zhou et al. encapsulated tumor-specific T cells within the outer layer of pancreatic tumor organoids to mimic the physical barrier, thus summarizing immune infiltration within the immunosuppressive TME [45]. Currently, a novel 3D hydrogel has been developed to emulate the mechanical characteristics of intestinal tissue. This hydrogel accommodates patient-derived intestinal organoids and peripheral blood mononuclear cells (PBMCs), effectively replicating several crucial immune processes observed in vivo. These processes include bystander signaling, immune cell migration, and immune cell infiltration. Leveraging this co-culture model has allowed for the elucidation of the mechanism behind T-cell bispecific antibody-mediated immune cell activation and the subsequent cascade of off-target toxicity [46]. Cancer immunotherapy has introduced a novel treatment approach known as Chimeric Antigen Receptor T-cell therapy. This method involves enhancing patients’ T cells to better recognize and effectively target cancer cells, thereby facilitating their eradication [47]. The simulation of the in vivo microenvironment to evaluate the antitumor activity of Lewis Y antigen (Le_Y_)-specific chimeric antigen receptor (CAR) T-cells using prostate cancer organoids was successful [48].

### Multicellular platforms

In vivo, the intricate interplay between the tumor and its surrounding microenvironment forms an “ecosystem” that influences nearly every stage of tumor development and progression. This ecosystem encompasses various processes, ranging from basic gas exchange and transport of soluble factors to more complex alterations in physicochemical properties like acidification and hypoxia [8, 49]. From tumor neovascularization to the intricate cascade of intravasation, circulation, and extravasation involved in metastasis, it becomes evident that the tumor does not exist in isolation [8]. Therefore, the establishment of a multicellular, multi-parameter in vitro cancer model may provide an alternative platform that better simulates the in vivo TME.

#### The air-liquid interface (ALI)

The air-liquid interface (ALI) organoid culture method offers an ideal experimental setting for studying the interaction between exogenous compounds and cells. This is due to its unique features, including surface exposure and substrate nutrient supply, facilitating a closer examination of these interactions [50, 51]. This method facilitates the construction of organoid models comprising a variety of immune cells, making it well-suited for advancing immuno-oncology research within the TME. Initially applied by Calvin J Kuo et al. [52] to the study of continuous proliferation and multilineage differentiation of primary mouse intestine, the ALI method has been further validated for its efficacy. Using the ALI method, various types of tumor cells can typically proliferate and generate organoids while preserving the pathological features, genetic diversity, and intricate stromal histological architecture of the original tumor, including functional tumor infiltrating lymphocytes (TILs). In addition, Laura K. Esser [53] et al. demonstrated that the ALI model can effectively mirror the varied treatment responses of clear cell renal cell carcinoma in distinct individuals.

The ALI approach offers an important advantage in preserving epithelial-immune cell interactions while also allowing for precise control of oxygen concentration at the culture interface through gas composition adjustments. Leveraging the benefits of collagen for facilitating cell ventilation and polarity, the ALI method has additionally been employed to create epithelioid sarcomas exhibiting both epithelial and mesenchymal cell morphology and immunophenotypes [54, 55]. Nonetheless, the ALI method has certain limitations when studying the immune microenvironment, primarily due to the susceptibility of immune components to decay, leading to transient cell interactions [52, 56]. Hence, while the ALI method exhibits significant promise in constructing personalized tumor organoid models, further development and refinement of its methods and applications are warranted.

#### Microfluidic chip

Incorporating tumor organoids into Microfluidic Chip devices facilitates the creation of multi-cellular, multi-parameter simulated TME platforms. This enables flexible customization of chamber structures and channel combinations to suit experimental requirements. The goal is to achieve precise spatial control over tumor organoids, cell distribution, and fluid flow rates [57]. Nowadays, tumor organoids combined with microfluidic chips have been widely used in TME research [58]. Haque et al. developed a multicellular microfluidic chip termed PDTO, integrating stromal cells such as pancreatic stellate cells and macrophages. This model not only extended cell function and lifespan but also effectively simulated a complex tumor milieu comprising organoids with fibrotic stroma and immune cells. Furthermore, the model demonstrated that matrix-targeted therapy markedly augmented the cytotoxic impact of chemotherapeutic agents on tumor cells. These findings underscore the promising utility of tumor chip devices in evaluating drug efficacy [59, 60]. Moreover, Strelez et al. illustrated that tumor organoids carrying KRAS mutations display heightened sensitivity to mechanical stimuli within the TME by leveraging fluidic and mechanical stretching functionalities. They also utilized gamma-aminobutyric acid, a neurotransmitter, as an energy source to augment their invasive tendencies [61].

In addition to tumor matrix research, the multi-cellular platform constructed by combining organoids with microfluidic technology can also be used as an effective platform to reconstruct the immune microenvironment [62]. Utilizing microfluidic chip technology to co-culture mesenchymal stem cells (MSCs), PDTOs, and PBMCs revealed several significant outcomes. Firstly, allogeneic MSCs significantly enhanced the survival rate of PDTO cultures and concurrently accelerated the growth rate of PDTO samples. Furthermore, MSCs facilitated the differentiation of monocytes into tumor-associated macrophages, effectively recapitulating the immune microenvironment akin to the primary tumor [63]. Furthermore, a flow condition platform was constructed, comprising endothelial tubes, pancreatic stellate cells, and pancreatic ductal adenocarcinoma (PDAC) organoids, to investigate the role of the TME in facilitating immune cell recruitment and modulating their interaction with pancreatic cancer cells. Research has revealed that stromal cells within the TME can establish a physical barrier, impeding the migration of immune cells towards cancer cells and providing a protective shield for cancer cells. Simultaneously, the TME generates a distinct biochemical milieu that appears to attract immune cells and influence their distribution and functionality within tumors [64]. The construction of a complex tumor microenvironment containing fluid dynamics and immune cell infiltration can be achieved through the application of microfluidic chip technology, enabling a more realistic simulation of the growth state of tumors in vivo. The method of combining microfluidic systems with tumor organoids has significantly expanded our understanding of the TME. Integrated platforms of this nature are rapidly gaining importance as well as diverse functionality in the construction of cancer models and the study of tumor-cell matrix interactions [65, 66].

### Bioprinting

Bioprinting can precisely arrange active biomaterials and living cells, form structures with bionic morphology, and meet the needs of specific cellular micro-environment and biological functions, thereby creating complex and functional 3D biological structures. This suggests the potential for utilizing 3D bioprinting to create organoid culture systems encompassing intricate environments suitable for diverse tumor types [26, 67, 68]. In 2020, Professor Kunyoo Shin’s group introduced a new concept of advanced mini-organs called bladder assembloids using 3D biopanning technology. These assembloids combine normal bladder stem cells or organoids derived from bladder cancer patients with tissue matrix and other components in the micro-environment, including fibroblasts, endothelial cells, immune cells, and muscle cells, to form composite tissues [69]. A recent study combined biomimetic bioink and 3D bioprinting technology to print a highly simulated vascularized LCO (lung cancer organ) model by combining LC (lung cancer) cells, lung fibroblasts, and pulmonary decellularized extracellular matrix (LudECM). This model effectively replicates the vascular perfusion system, tissue-to-tissue interface, and tissue-specific structural features at the level of TME, demonstrating significant advantages in simulating real biological systems [70]. The fusion of tumor organoids and 3D bioprinting technology is demonstrating tremendous promise and practical value in simulating the TME [71]. Nevertheless, to advance the widespread adoption of this field, numerous hurdles must be surmounted. These include refining material selection, overcoming technical barriers, enhancing model authenticity, and transitioning to clinical application. The resolution of these challenges will bring progress for tumor models to more accurately reflect the situation in the human body, thus promoting the development of personalized medicine and precision treatment strategies [26, 72].

## Single-cell sequencing and spatial profiling

### Computational biology

The fusion of tumor organoids and 3D bioprinting technology is demonstrating tremendous promise and practical value in simulating the TME. The inclusion of genetically diverse sample donors is essential in oncology research to assess individual differences between populations [73]. To maximize the potential of tumor organoid models, rigorous characterization and validation have become essential [74, 75]. Therefore, once the patient-derived organoid model is successfully constructed, it requires further analysis and processing. Beyond traditional methods like morphological and immunohistochemical identification, multi-dimensional data integration at the molecular profile level can be performed on tumor organoids. Advanced technologies such as next-generation sequencing, single-cell sequencing, and spatial omics offer powerful analytical tools for evaluating these organoid models [76–78]. Comparing the gene profiles between tumor organoids and matched ex vivo tumor samples, can not only guide the development of organoids but also enhance their further application in drug testing, disease mechanism research, and other fields, thereby providing a strong scientific basis for personalized medicine and precision medicine [79]. Establishing a tumor organoid biobank serves as an effective enhancement to the establishment of a sustainable biobank. Once the biobank is successfully established, its samples can be thoroughly characterized through an integrated approach combining genomics, transcriptomics, proteomics, and single-cell sequencing. This not only elucidates the consistency between the histological and molecular characteristics of the tumor organoids and their corresponding parental tumors but also gathers various tumor subtypes within the library. Moreover, it demonstrates the gene expression patterns of cells in the tumor organoids and enhances our understanding of their functional status in specific environments [14, 73, 80]. Combined with high-throughput drug screening, pharmacogenomic analysis can reveal the different response patterns and potential drug genomes of different molecular subtypes of tumor organoids under the gene-drug association mode. This offers a novel perspective and scientific foundation for predicting drug responses, aiding in the development of potential personalized treatment strategies [81, 82].

**Fig. 3:**
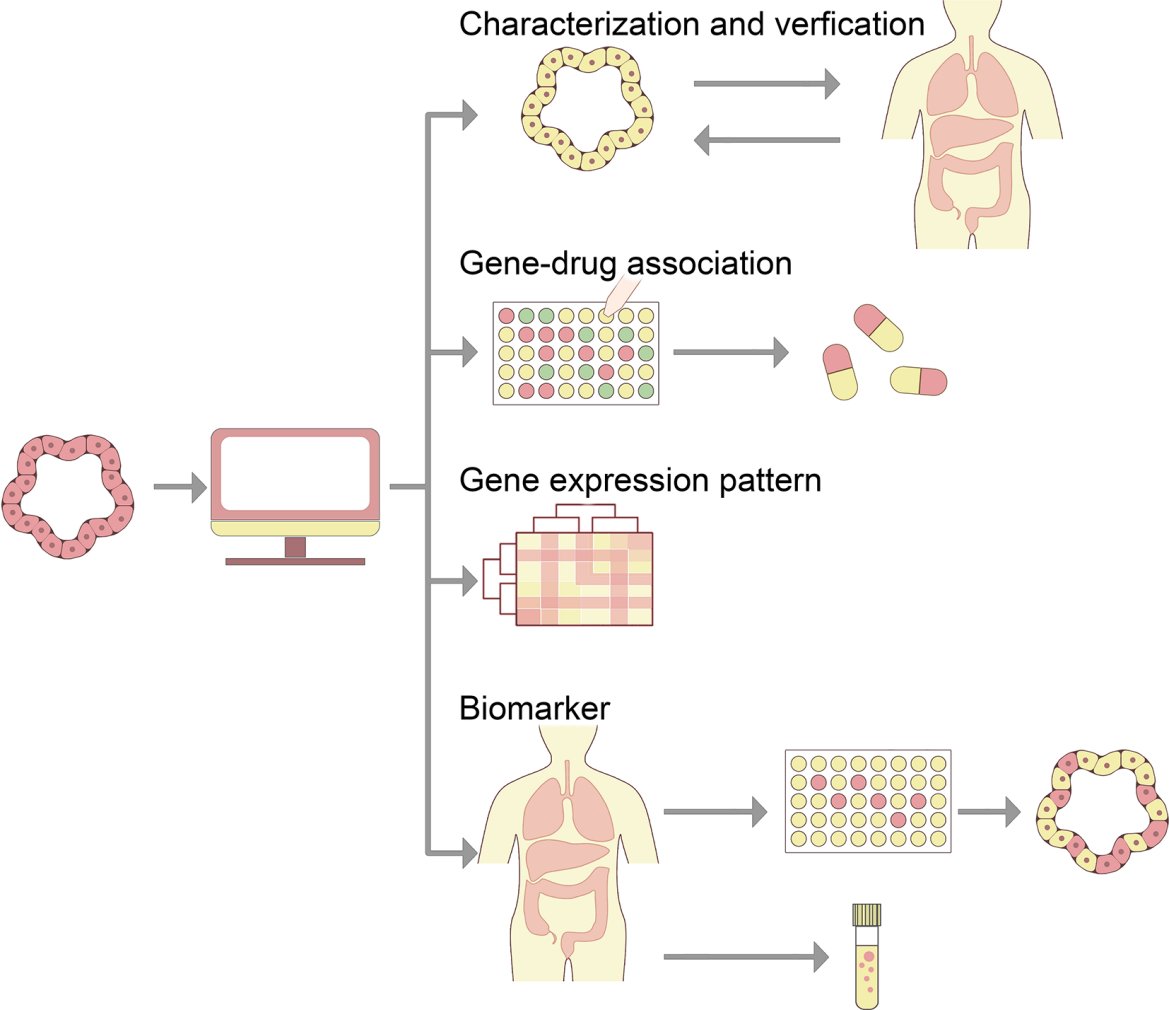
A data model of tumor organoids is constructed to comprehensively characterize the relationship between tumor and model, disease biomarkers and potential therapeutic targets are identified, and cell behavior and drug response are predicted

The vast amount of multi-omics data presents a significant challenge to the continuity of organoid research. Integrating multi-dimensional data allows for a comprehensive evaluation of tumor organoids through statistical analysis and computational modeling across various biological molecular levels [83, 84]. The integration of tumor organoids with multi-dimensional data not only improves the quality control of organoids but also offers a reference framework for the application and research of organoids at the data level [85, 86]. For instance, Sanguk Kim and colleagues identified robust translational drug biomarkers by analyzing pharmacogenomics data from colorectal and bladder cancer organoids, combined with network analysis. This approach supplements existing methods for predicting drug response in cancer patients at the data level [85]. In addition, Shi et al. [87] identified the key transcription factors across different molecular subtypes of pancreatic cancer by integrating data from chromatin accessibility maps, whole genome sequencing, transcriptome sequencing, and epigenetic-related chemical and drug sensitivity analyses. They also predicted key chromatin accessibility peaks closely linked to gene regulatory networks. Further, a potential cancer driver mutation in the regulatory non-coding region was found. In summary, the application of multi-dimensional data integration technology significantly expands the depth and breadth of organoid research, encompassing dynamic tissue and cell analysis as well as gene and molecular level exploration (Fig. 3).

### Organoid atlas

The data continues to proliferate, and the comprehensive molecular profiles associated with organoids are forming an extensive tree of life. Under the European Union Horizon 2020 initiative, the “HCA | Organoid” project (https://HCA-Organoid.eu) aims to achieve the core objectives of the Organoid Cell Atlas. This project seeks to verify the feasibility and application potential of combining human organoids with single-cell and spatial genomics data. By integrating these advanced single-cell technologies, the project aims to more accurately depict the complex biological characteristics of human organoids, thereby advancing the development of human disease models and deepening our understanding of disease mechanisms [73, 88]. DISCO is an in-depth integrated human cellomics data database (https://www.immunesinglecell.org/), which also covers the efficient integration of class organ single-celled omics data analysis. It aims to explore cellular complexity and heterogeneity by aggregating and standardizing single-cell data in the field of organoids [89]. Further, OrganoidDB (http://www.inbirg.com/organoid) is a specialized database that provides a critical tool for the multi-faceted exploration of batch and single-cell transcriptome studies. This resource promotes the generation of organoid models that closely resemble living organs in composition, structure, and function [90]. The advent of these datasets will offer essential information to support the complex regulatory networks involved in organoid development and differentiation, thereby enhancing the application potential of organoids in precision medicine and regenerative medicine.

## Tumor organoids for precision medicine

PDTO was originally developed to model tumor phenotypes in vivo and study cancer progression under ex vivo conditions. In contrast to traditional cancer cell line models, PDTO models can more faithfully preserve the genetic diversity and phenotypic heterogeneity of tumors in vivo. This capability overcomes the limitations posed by the genetic instability often observed in cancer cell lines. This superiority has been validated through numerous morphological and genetic profiling analyses [91, 92]. Furthermore, while PDTX can also maintain the histopathological and genetic traits of their parent tumors, the construction time required for PDTO models is shorter—measured in weeks rather than months. This substantial reduction in construction time significantly shortens the cycle for establishing in vitro model platforms relied upon for drug testing [10]. The capacity of PDTO models to mimic various tumor subtypes, encompass disease heterogeneity and undergo controlled construction renders them a potent preclinical analytical instrument for forecasting drug reactions in vivo and delving into cancer progression [92]. Based on these characteristics, PDTO models provide an effective means to overcome several limitations in tumor drug testing research.

Currently, the sensitivity of numerous chemotherapeutic and targeted drugs has been evaluated utilizing a single tumor organoid model, offering valuable insights for clinical decision-making [93]. For instance, the HOPE (Harnessing Organoids for Personalized Therapy) trial in PDAC patients has been executed to assess the viability of cultivating PDTO directly from PDAC patient samples in real-time. Moreover, the trial aims to categorize PDTOs based on their responsiveness or resistance to treatment protocols. Through this study, we prospectively examined the association between PDTO and the response of donor patients to traditional anticancer medications by forecasting the clinical and analytical factors conducive to successful PDTO cultivation. Based on these findings, researchers have devised a series of predictive models to gauge the effectiveness of disease control [94]. This approach not only showcases the potential of tumor organoids in prospective treatment assessment but also charts a new course for enhancing the precision and efficacy of treatment protocols. Furthermore, through the comparison of PDTO, PDTO-derived xenograft responses, and actual patient responses to anticancer drugs in clinical trials, it has been discovered that PDTO subjected to anticancer treatments not only undergo further validation in PDTO-based xenograft models but also effectively recapitulate patient responses to treatment in clinical trials [95, 96]. Moreover, through the analysis of the gene expression profiles of organ models derived from BTC patients exhibiting varied chemotherapeutic responses, the researchers have successfully identified and established a gene expression panel capable of predicting the response of BTC patients to chemotherapy drugs [96]. During treatment, it’s common for patients to exhibit inadequate responses or develop drug resistance. At such junctures, the utilization of PDTO offers a groundbreaking functional precision medicine approach to the clinical application of in vitro drug testing for patients who have failed to respond to standard care treatments. For example, GRAY [97] et al. employed tumor organoids to conduct drug sensitivity analysis, identifying targeted therapy for a patient with platinum-resistant advanced low-grade serous ovarian cancer across various stages of resistance. Subsequently, the patient experienced a significant improvement in their condition post-treatment. This approach not only expands the array of potential drug options for patients unresponsive to standard treatment protocols but also markedly enhances the precision of personalized treatment. Furthermore, it highlights the invaluable contribution of PDTO in research aimed at overcoming drug resistance.

To date, researchers have utilized various types of tumor organoids to investigate drug responses across different stages of patient treatment (Table 2), thereby enhancing the functionality of tumor organoids. Several studies have consistently affirmed the high degree of consistency between a patient’s primary tumor and the corresponding PDTO, retaining morphology and genomics. These insights furnish a robust tool for preclinical investigations, enabling accurate predictions of drug sensitivity in vivo and deeper exploration into the mechanisms driving cancer progression [92, 98].

**Table 2:** By testing how different tumor organoids respond to various treatments, researchers can identify the most effective therapies for specific cancer types

Types of PDTO	Tissue source	methods	High throughput screening	Reference
Follicular lymphoma	Patient	RNA sequencing	Patient-specific modeling, high-throughput screening, TME feature recognition, and therapeutic response assessment were achieved by cultivating patient-derived lymphoma organoids (PDLO)	[135]
Pancreatic ductal adenocarcinoma	Mice	High throughput screening	In combination with CRISPR-Cas9 technology, developing isogenic mouse pancreatic organoids containing common PDAC driving mutations to screen drugs through a high-throughput drug screening platform	[148]
Metastatic colorectal cancer	Patient	Whole genome sequencing and drug screening	Using a standardized machine detection platform for organoids to predict drug response	[149]
Locally advanced rectal cancer	Patient	Short tandem repeat detection	Developing a patient-derived tumor organoid (PDTO) platform to predict postoperative adjuvant chemotherapy (AC) effects in patients with locally advanced rectal cancer (LARC) who do not respond well to neoadjuvant chemoradiotherapy.	[150]
Non-small cell lung cancer	Patient	Multiparameter analysis	Using a multi-parameter analysis method (Prediction of diagnostic reactivity of Cancer organoids based on CODRP) to perform drug susceptibility tests on organoids from patient tissues and confirmed the primary cancer features of PDO by pathologic comparison of tissue sections	[79]
Mucosal melanoma	Patient	Whole exome sequencing and transcriptome sequencing analysis	Using OMM organ tissues to explore a faithful model of tumor evolution and immunotherapy combination strategies, study drug response and explore treatments in combination with anti-PD-1 therapy	[151]
Adenoma of colon rectum	Patient	High throughput screening	Establishing patient-derived high-risk colorectal adenoma organoids (HRCA-PDO) biobank and conducted a series of high-throughput, high-content HRCA drug screening	[106]
Vascularized lung cancer	Patient	3D printing, co-culture of tumor organoids and stromal cells	Evaluating drug reactivity in a vascularized LC model simulating pulmonary fibrosis	[70]
Low grade serous ovarian cancer	Patient	Whole exome sequencing and high throughput screening	Using organoids from patients’ tumors for drug sensitivity analysis and then personalize treatment	[97]
Pancreatic ductal adenocarcinoma	Patient	Whole exome sequencing	Constructing the amplified biomass of patient-derived tumor organoids to improve sequencing quality and predict clinical treatment response	[144]
Heterogeneous colorectal cancer	Patient	Whole exome sequencing	Constructing a heterogeneous colorectal cancer PDO biobank and a large-scale functional screening of double-targeted bispecial antibodies (bAbs) on paired healthy colon mucosal samples	[152]
Colorectal cancer	Patient	Immunohistochemical (IHC) staining, flow cytometry, drug screening	Identifying four drug candidates for colorectal cancer treatment by screening colorectal cancer organoids to determine individualized treatment regimens and predict drug responses to neoadjuvant therapy	[153]
Endocrine resistant, refractory and metastatic breast cancer	Patient	Proteomic analysis	Isolating the preclinical drug sensitivity of CTCs generating organoids to predict metastasis from an orthotopic CRC xenotransplantation model	[154]
Upper urinary tract urothelial carcinoma	Patient	Xenotransplantation and immunohistochemistry	Constructing an PDO model to explore the mechanism of low energy shock wave (lesw) and cisplatin combined therapy for UTUC	[155]
High-grade serous tubo-ovarian cancer	Patient and mice	Gene editing and RNA sequencing	Constructing a high-grade serous tubal ovarian cancer (HGSC) model to develop a chemotherapy/immunotherapy regimen that produces a persistent T cell-dependent response in HGSC	[156]
Pancreatic ductal adenocarcinoma	Mice	Whole genome sequencing and transcriptome sequencing analysis	Constructing PDOs analytical genomics, transcriptomics, and therapeutic profiles to identify molecular and functional subtypes of pancreatic cancer and predict therapeutic response	[99]
Triple Negative Breast Cancer	Patient	Xenotransplantation, morphological analysis, whole genome DNA methylation analysis, and exome sequencing	Using PDXs and derived organoids (PDXOs) for drug screening and in vivo validation	[157]
Pancreatic ductal adenocarcinoma	Mice	High throughput screening	Using wild-type (WT) or isogenic mouse pancreatic organoids containing common PDAC driving mutations, we constructed a high-throughput drug screening platform to screen and identify that cyhexacillin malexate can inhibit growth and induce cell death in Kras G12D mutated pancreatic organoids	[148]
Prolactin pituitary neuroendocrine tumors	Patient	Transcriptome sequencing analysis and single cell sequencing analysis	Conducting transcriptomic sequencing and single-cell sequencing analysis for DA resistant prolactin adenomas and sensitive prolactin adenomas, constructed organoid models for drug screening, and evaluated the effectiveness of identified drugs in vitro and in vivo	[131]
Head and neck squamous cell carcinoma	Patient	RNA sequencing	Constructing an organoid model to validate the cytotoxicity and sensitization potential of chemoradiotherapy targeting 10 RTK and β1 integrins and screened the drugs	[158]

## Imaging and analysis of organoids

Currently, organoids are widely acknowledged as precise in vitro tools for drug susceptibility testing. Common evaluation methods employed in drug sensitivity assessments using tumor organoids include IC50, area under curve (AUC), and growth curve analysis, among others, which represent the mainstream approaches in organoid drug research [99, 100]. In conducting these studies, researchers frequently confront the following challenges: (1) Testing multiple drugs necessitates swiftly preparing sufficient tumor organoids in large quantities to assess the varied killing effects of different drug concentrations on tumor cells. (2) Drug sensitivity analysis using PDTO often lacks multidimensional evaluation methods, presenting a relatively singular dimension. (3) Despite employing the same experimental protocol, data variability may arise due to the absence of standardized operating procedures, along with differences in reagents, instruments, and data analysis methods [101, 102].

Fortunately, these challenges have attracted the attention of many researchers. To address these challenges, many innovative concepts have been proposed to improve the efficiency of organoid model preparation and optimize analytical methods to enhance data consistency and reliability [103]. For high-throughput analysis of organoids, Celos, An analytical tool, offers a high-precision, high-throughput analytical pipeline. It employs Stardist-3D convolutional neural networks for 3D organoid segmentation and kernel segmentation. This approach allows for the recapitulation of traditional luminescent drug response testing. Furthermore, Celos enables quantitative analysis of the morphological changes in organoids and nuclei induced by treatment. It also facilitates the assessment of the spatial relationship between cells exhibiting ecological affinity [104]. At the same time, a high-throughput automated live cell image analysis software called Organoid Brightfield Identification-based Therapy Screening (OrBITS) compatible software has been designed. By integrating computer vision technology with convolutional neural network machine learning methods, we can identify the optimal experimental parameters and drug response indicators. This not only aids in refining patient stratification but also enhances the precision and consistency of drug response quantification in organoid models [105]. In addition, high-content fluorescence microscopy imaging technology can perform accurate segmentation and quantitative analysis of individual organoids and nuclei in organoid models. This approach effectively discerns the effects of cytostatic and cytotoxic treatments, offering a crucial supplement to drug research. It not only compensates for potential biological information gaps in tumor organoid-based studies but also unveils potential heterogeneity among organoids. This allows researchers to more accurately assess the drug impact on specific tumor organoid models, thus laying a solid foundation for developing more effective treatment strategies [106–108].

With the development of artificial intelligence, the integration of AI and organoids can simplify the analysis and processing of complex datasets at the level of machine learning. The integration of AI and organoids enables the analysis and mining of cross-scale and multimodal data. When coupled with machine learning, it facilitates data mining tasks in multicellular systems and organoids [109]. MOrgAna is a Python-based software tool that integrates machine learning algorithms for the analysis and segmentation of organoid images. This software addresses challenges in image analysis by automating processes and ensuring data comparability across various imaging platforms through a set of reliable quantitative metrics. Such features facilitate a comprehensive understanding of the morphological characteristics and functions of organoids [110]. Another study has successfully developed an analysis tool called Phindr3D. Phindr3D uses data-driven machine learning methods to analyze cell phenotypes, with the ability to quickly and accurately examine complex biological culture systems. It effectively addresses the challenge of identifying the number of biologically relevant clusters in dataset analysis and accurately locates the volume of individual organoids within a large number of image stacks. This capability eliminates the necessity for traditional cell segmentation methods [111]. This not only expedites the data analysis process in the biomedical field but also significantly enhances the accuracy and efficiency of data interpretation. Consequently, it demonstrates the intricate interactions between cells and their functional interpretation at the tissue level.

## Organoids combined with gene editing for the development of tumors

**Fig. 4:**
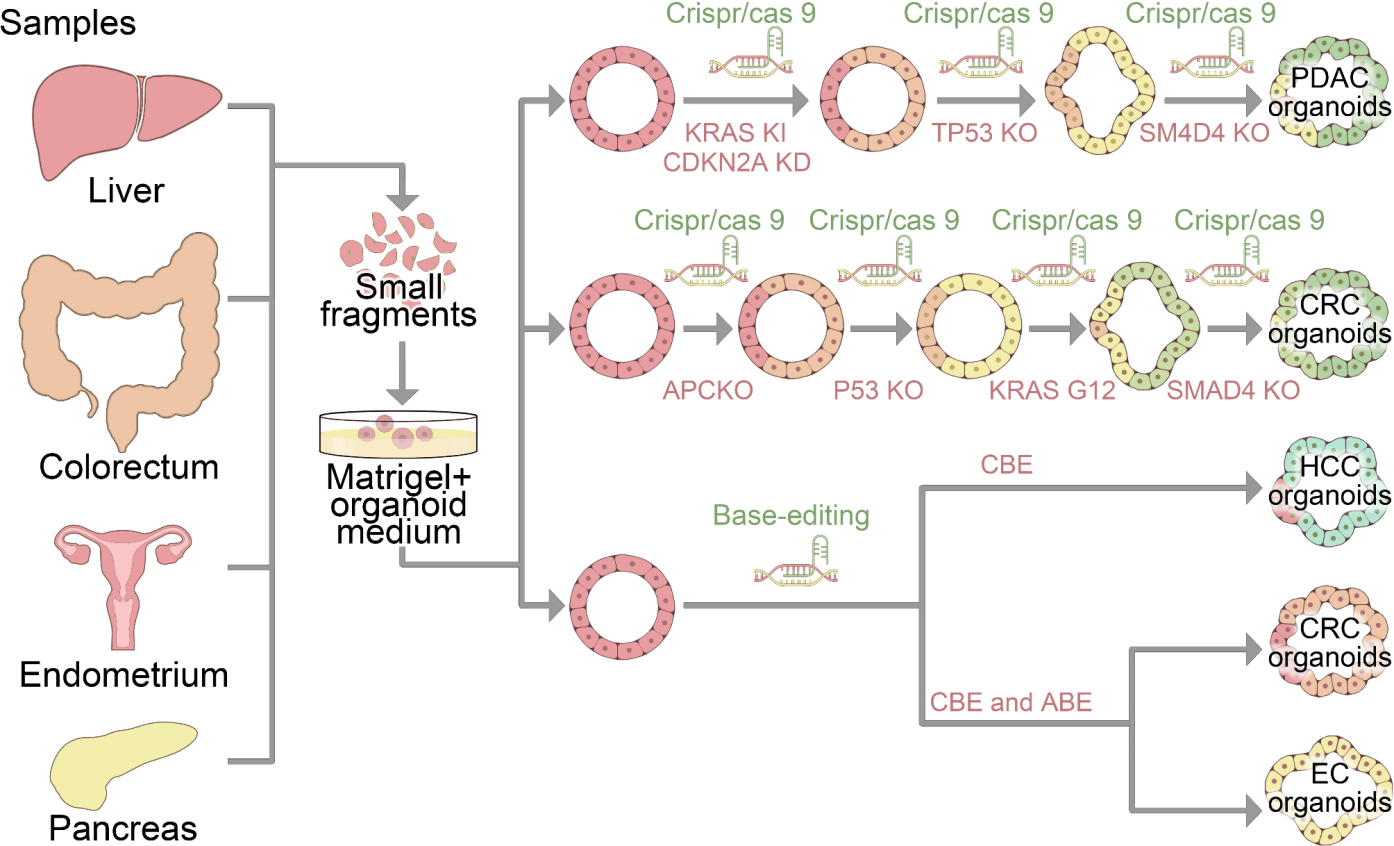
Gene Editing of Organoids Using Genome Editing Methods involves Crispr/cas9 editing technology and base editing technology for studying the occurrence of tumors and the development of tumor models

The evolution of cancer is the result of a series of orderly mutations in cancer-specific genes in normal cells [112, 113]. This aspect of cancer development, coupled with the emergence of genetic engineering technology, has facilitated the creation of genetically well-defined cancer models. With the capability for gene editing in epithelial-derived organoids, researchers can employ normal epithelial cells to conduct in vitro simulations and mutational analyses at different stages of malignancy. This approach serves to deepen our comprehension of natural carcinogenesis [114]. The early adoption of Crispr/Cas9 gene editing technology offers significant assistance in generating tumor organoids with specific genetic backgrounds [115, 116]. For example, Takashi Seino et al. started with normal human pancreatic organoids and successfully generated pancreatic ductal adenocarcinoma characteristic organoids in vitro by inducing KRAS G12V mutations and loss-of-function mutations in CDKN2A, TP53, and SMAD4 genes (Fig. 4) [117]. Similarly, Dorst et al. sequentially edited four tumor-related genes—APC, p53, Kras, and SMAD4—in human intestinal stem cells. This led to the generation of quadruple mutant organoids harboring both activating mutations in KrasG12D and inactivating mutations in APC, p53, and SMAD4. Upon implantation into the cecum of mice, these quadruple mutant organoids displayed aggressive features akin to colorectal cancer. Additionally, the loss of APC and p53 was adequate to induce chromosome instability (CIN) and facilitate spontaneous metastasis of the orthotopically located tumor to the liver and lungs of mice, without any external intervention (Fig. 4) [118]. It is worth mentioning that the engineered organoids obtained by gene editing can form tumors when implanted in xenografts, but the tumors formed do not show high metastatic potential, and large-scale metastasis still requires the acquisition of CIN [119, 120].

As a revolutionary gene editing tool, Crispr/cas9 technology has great potential in the development of organoid models. Using Crispr/cas9 and other gene editing technologies, the cells in organoids can be modified to achieve specifically related genes or biological pathways. However, there are several challenges associated with the application of Crispr/Cas9, including the under-specificity of the target, low editing efficiency, and the potential risks to genome stability. These issues primarily stem from the Crispr/Cas9-dependent DNA double-strand break mechanism. This mechanism can lead to the mispairing of the designed single-stranded guide RNA (sgRNA) with non-target DNA sequences, resulting in off-target effects and the introduction of unexpected gene mutations [121, 122]. The Base Editing technique developed by Liu et al. [123] effectively circumvents the issue of non-specific editing associated with DNA double-strand breaks (DSBs) by enabling the replacement of a single base. Moreover, traditional Crispr/Cas9 technology requires enhancement in editing efficiency, particularly for inducing multiple gene mutations in organoids to construct tumor models with specific genetic backgrounds. This process is inherently time-consuming [124, 125]. Professor Hans Clevers and his team utilized base editing technology to create both adenine base editor (ABE) and cytidine base editor (CBE) for constructing cancer models in organoids derived from human adult stem cells. Employing a multiple-gene editing composite strategy, the team introduced several cancer-related single-base mutations into the organoids in a single step through the co-electroporation method. This approach successfully generated tumor models that accurately mimic hepatocellular carcinoma, endometrial cancer, and colon cancer [126]. The high flexibility and timeliness of this method render it an ideal tool for simulating cancer across various epithelial tissues, significantly broadening the application prospects of in vitro solid tumor research models (Fig. 4).

### Modelling of special types of organoids

Although most of today’s tumor organoids are of epithelial origin, there are also many special types of non-epithelial organoids, which is a noteworthy development and expands the application of organoids in cancer research. Employing human IPSCs to generate bone marrow organoids (BMOs) aligns with this concept, enabling the emulation of the human bone marrow microenvironment. This approach opens up a multitude of research and application possibilities [127]. A BMOs model capable of culturing bone marrow and primary cells from patients with lympho-hematological cancer has received attention. This model accurately replicates the niche and hematopoietic components of specific species and cell environments. It not only simulates hematopoietic microenvironment interactions under physiological conditions but also supports cell transplantation and survival in patients with hematological tumors. This advancement fulfills the pressing demand for 3D human bone marrow models in translational research [128]. In another study, a patient-derived melanoma organoid model was constructed by using air-liquid interface culture technology and the matrigel culture method. Researchers utilized MPDOs to assess the tumoricidal of immunotherapies and investigated the potential of small-molecule compounds to augment the effectiveness of immunotherapies [129]. In addition, retinoblastoma [130] and prolactinoma [131] organoid models have been successfully constructed, maintaining the genomic properties of the source tumors in the process. By conducting drug screening experiments on these organoid models, the efficacy of the identified drugs can be evaluated in vitro. The ongoing development of these specialized organoids signifies a growing understanding of the tumor genesis, progression, and treatment mechanisms of non-epithelial cells in oncology research.

### Summary and prospect

Oncology research is currently undergoing a revolutionary transformation, particularly in the new era marked by the integration of oncology and organoid technology. Tumor organoids have emerged as a novel model in scientific inquiry, significantly advancing the understanding and development of complex cancer models [132]. During the evolution of organoid technology, patient-derived organoids can simulate the disease state of a specific patient, providing accurate models for studying disease mechanisms and treatment responses. Hence, PDTO holds immense potential for testing various drugs, assessing treatment efficacy and side effects, and tailoring the most suitable treatment for patients [63, 133, 134]. Simultaneously various components of TME should be effectively integrated to build a more complete and accurate tumor simulation platform. While the current simulation of the TME using organoid models remains a “partial simulation”, there are ongoing challenges regarding the vascularization and matrix of tumor organoids. Nevertheless, as co-culture models, microfluidic technology, bio-printing, and other technologies steadily advance, researchers are hopeful regarding the complete replication of dynamically changing TME in vitro [43, 62]. Furthermore, the emergence of specialized organoids derived from non-epithelial cells offers a fresh perspective for gaining a deeper understanding of the biological characteristics of tumors [135]. The combination of advanced imaging techniques and computational biology is constantly pushing the boundaries of tumor organoid research [136]. The integration of these technologies not only enhances the precision and efficiency of analyzing tumor organoids but also equips researchers with a potent tool to observe the microstructure and cellular behavior of organoids in real-time. Green nanomaterials, synthesized sustainably, hold promising potential when combined with organoid applications. In drug delivery, nanoparticle-mediated methods can enhance tumor targeting and therapeutic efficacy, and have already been utilized in various treatments [137].For instance, Zheng and colleagues utilized organoid models to validate the concept of anti-Rspo therapy and the integration paradigm of nanoparticles for targeted cancer treatment [138]. Moreover, referencing the integration of cardiovascular biomaterials with autologous cell sources can offer patients safer and more natural tissue engineering techniques. This paves the way for constructing novel organoid-based biomaterials on a nanoscale to potentially replace or restore the function of injured or lost tissues and organs [139, 140].

While organoids have demonstrated significant potential in personalized medicine, there remains scope for enhancing the efficiency of construction, scale production, and cost control of tumor organoids. The rapid advancement of 3D printing, bioengineering, and microfluidic technology has opened up new avenues for the efficient, cost-effective, and high-throughput preparation of tumor organoids [141, 142]. In particular, the integration of microfluidic and 3D printing technologies enables rapid manufacturing and development of both normal organs and tumors. Through automation control, this approach ensures uniformity and controllability throughout the entire process, facilitating large-scale production [143]. In addition, using the large-scale genomic data analysis of tumor organoids, molecular markers of disease and potential therapeutic targets can be effectively identified [144]. The utilization of simulation experiments to predict cell behavior and drug response, coupled with the application of machine learning algorithms to extract valuable insights from intricate datasets, offers robust backing for precision medicine. These factors play pivotal roles in driving forward the advancement of tumor organoids research [145]. Hence, establishing a comprehensive database, gathering and analyzing extensive data from organoid models, fostering knowledge dissemination, and bolstering research collaboration have emerged as critical focal points requiring immediate attention and concerted efforts in the research domain.

Despite many challenges, the potential of tumor organoids in the research and application is substantial. With the continuous development of 3D culture technology, tumor organoids are expected to reproduce the 3D structure and micro-environment of tumors, thus providing a more accurate research platform for revealing the mechanism of tumor occurrence and development. In the future, tumor research will increasingly prioritize the development and refinement of research models to attain greater clinical relevance and predictive accuracy. As an emerging tool in the realm of cancer research, tumor organoids will facilitate a deeper comprehension of the intricate mechanisms underlying cancer. They will help unveil new horizons in cancer research and furnish a more direct scientific foundation for devising precision treatment strategies.

## Data Availability

Not applicable.

## Abbreviations

3D: Three-dimensional
ABE: Adenine base editor
ALI: The air-liquid interface
AUC: Area Under Curve
BMOs: Bone marrow organoids
CAFs: Cancer associated fibroblasts
CBE: Cytidine base editor
CIN: Chromosome instability
ECM: Extracellular matrix
IC50: Half maximal inhibitory concentration
IPSCs: Induced pluripotent stem cells
MSCs: Mesenchymal stem cells
PBMCs: Peripheral blood mononuclear cells
PDAC: Pancreatic ductal adenocarcinoma
PDO: Patient-Derived Organoid
PDTO: Patient-Derived Tumor Organoid
PDTX: Patient-derived tumor xenograft
TDCs: Tissue-derived cells
TILs: Tumor infiltrating lymphocytes
TME: Tumor microenvironment
